# Introns: the “dark matter” of the eukaryotic genome

**DOI:** 10.3389/fgene.2023.1150212

**Published:** 2023-05-16

**Authors:** Kaitlin N. Girardini, Anouk M. Olthof, Rahul N. Kanadia

**Affiliations:** ^1^ Physiology and Neurobiology Department, University of Connecticut, Storrs, CT, United States; ^2^ Department of Cellular and Molecular Medicine, University of Copenhagen, Copenhagen, Denmark; ^3^ Institute for Systems Genomics, University of Connecticut, Storrs, CT, United States

**Keywords:** intron, evolution, splicing, snRNA, spliceosome, eukaryotes, gene expression

## Abstract

The emergence of introns was a significant evolutionary leap that is a major distinguishing feature between prokaryotic and eukaryotic genomes. While historically introns were regarded merely as the sequences that are removed to produce spliced transcripts encoding functional products, increasingly data suggests that introns play important roles in the regulation of gene expression. Here, we use an intron-centric lens to review the role of introns in eukaryotic gene expression. First, we focus on intron architecture and how it may influence mechanisms of splicing. Second, we focus on the implications of spliceosomal snRNAs and their variants on intron splicing. Finally, we discuss how the presence of introns and the need to splice them influences transcription regulation. Despite the abundance of introns in the eukaryotic genome and their emerging role regulating gene expression, a lot remains unexplored. Therefore, here we refer to introns as the “dark matter” of the eukaryotic genome and discuss some of the outstanding questions in the field.

## Introduction

**FIGURE 1:**
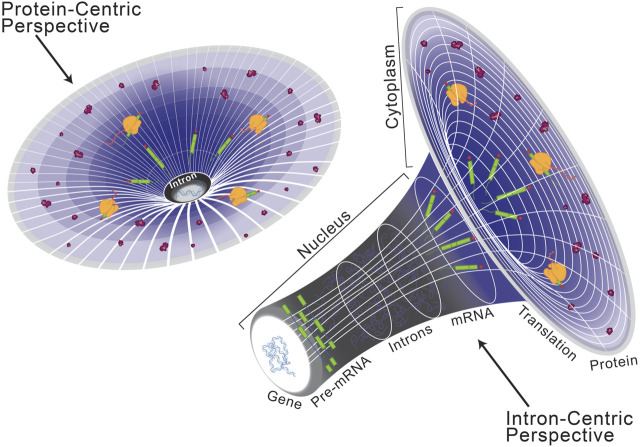
Schematization of a protein-centric versus intron-centric perspective on gene expression. Here, we model the role of introns in the genome after that of dark matter in astronomy, as both are difficult to characterize but critical organizing principles. From a protein-centric perspective (left), whereby the transcriptome and genome are interpreted in reference to a protein-coding sequence, it is easy to oversee the role of introns in eukaryotic gene expression. However, as depicted on the right, when the same model is viewed from an intron-centric perspective it becomes clear to see a regulatory mechanism by which introns are critical for expression of the eukaryotic genome.

Historically, introns were considered the non-coding, non-functional sequence elements which disrupt those that are protein-coding, called exons (Gilbert, 1978). While this protein-centric definition of introns (Figure 1, left) has served its purpose, their presence in long non-coding RNA reveals that introns are not specific to protein-coding genes but instead serve a broader role in eukaryotic gene expression (Krchňáková et al., 2019; Abou Alezz et al., 2020). Moreover, introns have been found to host other lariat-derived RNAs, including microRNAs, long noncoding RNAs, small nucleolar RNAs, small nuclear RNAs, and circular RNAs that are crucial for gene regulation (Liu and Maxwell, 1990; Hesselberth, 2013; Seal et al., 2020; Kumari et al., 2022; Vakirlis et al., 2022). Introns can also house enhancer elements that drive tissue-specific expression kinetics during complex vertebrate development and embryogenesis (Emera et al., 2016; Blankvoort et al., 2018; Meng et al., 2021; Shiau et al., 2022). These intervening sequences necessitated co-evolution of splicing machinery to facilitate production of a contiguous transcript capable of encoding a functional unit (Grabowski et al., 1985; Nilsen, 2003). Inhibition of splicing results in retention of introns in the mature transcript, which often disrupts the open reading frame and ultimately dictates the fate of the final transcript (Kaida et al., 2007; Effenberger et al., 2017; Olthof et al., 2021). Since the discovery of splicing, introns have been extensively investigated and the significance of splicing in regulating gene expression is well documented (Singh and Padgett, 2009; Tellier et al., 2020; Zhu et al., 2020; Agirre et al., 2021; Reimer et al., 2021). Taken together, the presence of introns has a significant impact on eukaryotic gene expression and underpins many of the complexities required to build higher eukaryotes. Therefore, here we present an intron-centric perspective (Figure 1, right) towards understanding regulation of eukaryotic gene expression.

## Function and evolution of intronic elements

Introns date back to the last eukaryotic common ancestor, after invasion into the early eukaryotic genome (Russell et al., 2006; Carmel et al., 2007; Csuros et al., 2011). While an endogenous model has been proposed to explain the emergence of introns (Catania et al., 2009), there is a general consensus that prokaryotic group II self-splicing introns underwent invasion and mutational degeneration during early eukaryogenesis, resulting in inert introns and trans-acting splicing machinery (Michel et al., 1989; Sharp, 1991; Sontheimer et al., 1999; Shukla and Padgett, 2002). As the origin of eukaryotic introns has been extensively described (Koonin, 2006; Rogozin et al., 2012; Vosseberg and Snel, 2017; Baumgartner et al., 2019; Smathers and Robart, 2019), here, we focus on the continued maintenance and diversification of introns in eukaryotic genomes.

**FIGURE 2:**
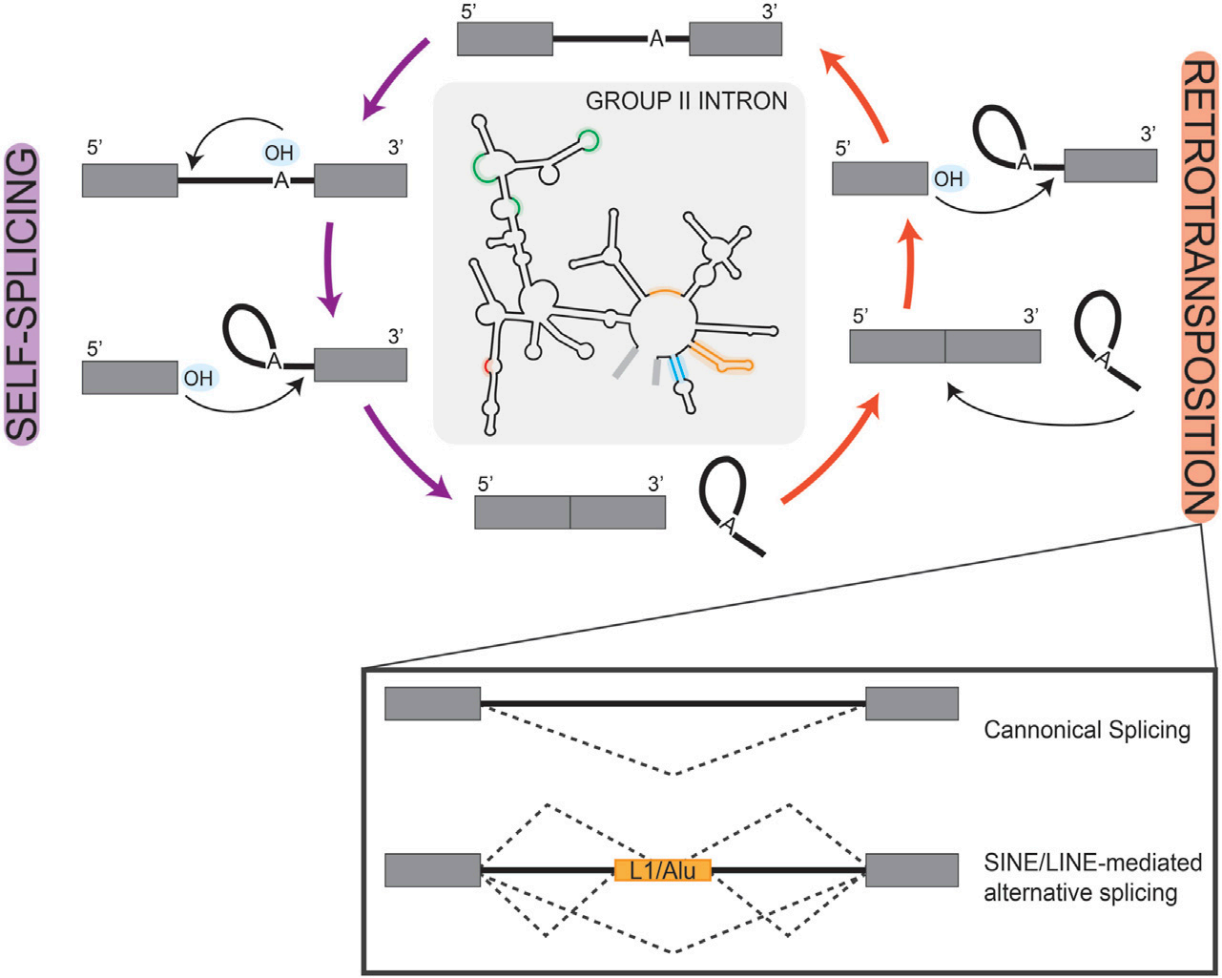
Retrotransposition of Introns. Simplified schematic of the reciprocal self-splicing (left, purple arrows) and retro-transposition (right, orange arrows) mechanisms that underlie the processing and mobility of group II self-splicing prokaryotic introns. These mechanisms are depicted as cyclic to highlight the parallel reactions that underlay each process. In the center, we show a group II self-splicing intron, with highlighted regions to represent loci that are analogous to eukaryotic snRNAs. In the box inset under retrotransposition, we show splicing schematics depicting the consequences of transposon-mediated alternative splicing in a eukaryotic gene. Here, boxes are used to represent exons and solid lines represent introns; splice patterns are represented by dashed lines.

Prokaryotic group II self-splicing introns behaved largely as transposable elements, which may have facilitated their invasion of the eukaryotic genome (Figure 2) (Lambowitz and Zimmerly, 2011)*.* Initially characterized in the maize genome, transposable elements are repetitive sequences found across eukaryotes and are critically known for their ability to relocate in the genome and alter gene expression (McClintock, 1950; SanMiguel et al., 1996; Elliott et al., 2005; Wells and Feschotte, 2020). Short and long interspersed retro-transposable elements (SINEs/LINEs) belong to the non-long terminal repeat class of elements which have retained transposable activity and are highly represented in the human genome as Alu and L1 elements, respectively (Kazazian and Moran, 1998; Lander et al., 2001; Balachandran et al., 2022). When carrying splice sites, these transposable elements can create novel exon/intron boundaries, which hold the potential to alter expression of that gene (Figure 2); a detailed description of exon/intron boundaries and their recognition by splicing machinery is discussed in the following sections. For example, a recent study queried pathogenic mutations that were associated with novel intron-exon boundaries in humans and identified those which aligned with transposable elements. They found that clusters of transposable elements are more liable to exonization, likely due to the combined effort of LTR and Alu elements in potentiating all necessary splice sites (Alvarez et al., 2021). In another computational investigation of the human genome, mutagenesis of Alu elements into weak splice sites was found to be well-tolerated if not retained long-term and was often associated with exon skipping events (Sorek et al., 2002). Exon skipping is a frequently observed form of alternative splicing, which more broadly serves as an important regulatory node for gene expression in developing systems (Baralle and Giudice, 2017). One can then speculate that Alu elements in this manner allow for transient sampling of novel functions of proteins encoded by these alternatively spliced transcripts. This idea is an extension of the already known role of Alu elements in tissue-specific transcription regulation (Franchini et al., 2011). Notably, weak splice sites in Alu elements can eventually become constitutively spliced exons, losing their capacity for transposition and become exons used in regulating tissue-specific gene expression, as is observed in the human *NARF* gene (Lev-Maor et al., 2007).

Inherent to the jumping nature of transposable elements is the impartiality of transposon landing. Transposon insertion would likely be deleterious in the protein-coding region of a gene, leading to evolutionary selection against that gene configuration. However, in a heterozygote, transposon-induced activation of a novel splice site within an intron could allow for a low-cost trial of differentially spliced isoforms, while still maintaining a functionally expressed copy. A susceptibility of spliceosomal introns to genomic recombination was demonstrated in two *Saccharomyces cerevisiae* genes, RPL8B and ADH2. Truncated versions of these genes were used in a splicing reporter construct, such that the second exon was expressed in frame with a fused EGFP cassette. Additionally, each construct carried an embedded *S. pombe his5* ^*+*^ gene within the first intron, encoded for in the opposite direction as EGFP. Here, the *his5* ^*+*^ gene contains an artificial intron lacking a catalytic branch point, and containing splice sites in such an orientation that they are only capable of splicing from the EGFP transcript. Thus, splicing of the artificial intron followed by transposition of the EGFP intron into the genomic loci was required to confer a positive result (Lee and Stevens, 2016). Meanwhile, Gozashti et al. (2022), has attributed rapid, lineage-specific intron gains to Introner elements derived from transposable elements. Through analysis of 1,700 species, these “intron-generating transposable elements families” were identified in approximately 5% of genomes and significantly overrepresented in aquatic lineages. Based on statistical association models and a consideration of likely propagation mechanisms, they concluded that Introner elements may facilitate recent intron gain, particularly through horizontal gene transfer in aquatic lineages. The activity of Introner elements is particularly interesting, as mechanisms of Introners in *Micromonas pusilla* and *Aureococcus anophagefferens* exhibit seemingly preferential insertion between pre-existing nucleosomes (Huff et al., 2016). The rationale here is such that the linker sequence between nucleosomes is often open and available for insertion events. Further support for this idea is seen in the unequal distribution and position of nucleosomes observed between protein-coding exons, pseudo exons, and introns in human and *Caenorhabditis elegans* (Andersson et al., 2009). Using transcriptomic and genomic sequencing data, Huff et al. (2016), reported that Introners are largely capable of co-opting splice sites and inserting by DNA transposition in both orientations, though with biases consistent with species-specific patterns in genome organization. Outside of splice site generation, transposons have also been implicated in regulation of splicing-competent snRNAs, such as those L1 transposons which are associated with formation of U6 pseudogenic snRNA (Doucet et al., 2015). Pseudogenes can encode variations of spliceosomal snRNAs, the implications of which are discussed further below. In all, transposable elements further expand gene structure by modifying intronic elements, thus revealing a critical role of non-coding intronic elements in eukaryotic genome evolution.

## Classification and splicing of introns

After the discovery of splicing, identified introns appeared to show a pattern of conserved terminal di-nucleotides at the exon-intron and intron-exon boundaries, and this feature became a defining characteristic of spliced introns (Breathnach et al., 1978; Crick, 1979; Breathnach and Chambon, 1981). As sequencing techniques have progressed and data now includes more diverse eukaryotic genomes, it is increasingly clear that introns are defined by several extended consensus sequences. These include the 5′ splice site (5′SS), the branch point sequence (BPS), and the 3′ splice site (3′SS) (Dietrich et al., 1997; Mercer et al., 2015). Not long after their discovery, it was determined that most introns are processed by five Uridylyl-rich snRNAs—U1, U2, U4, U5, and U6—that are highly conserved between eukaryotes and assemble into a ribonucleoprotein complex, the spliceosome (Bringmann et al., 1983; 1984; Bringmann and Lührmann, 1986; Nilsen, 2003; Wahl et al., 2009). Specifically, U1 snRNA has complementarity at the 5′ splice site, marking the exon-intron boundary, while U2 snRNA base pairs around a conserved adenosine toward the 3′ end, at what has become known as the branch point sequence (Yan and Ares, 1996; Malca et al., 2003). The direct base pairing of these snRNAs with splice site consensus sequences helps to recognize and remodel the intron during splicing, conferring the core function of the spliceosome.

As this mechanism was coming into focus, Jackson (1991), discovered spliced transcripts, that when mapped to the genome, showed intronic splice site sequences that were incompatible with the identified snRNAs. The fact that these introns were nonetheless spliced suggested the existence of a separate mechanism for their removal. This discordant finding led to sequence-based investigations for U snRNAs with complementary to non-consensus splice sites. This included an exploratory genomics investigation by Hall and Padgett (1994), and ultimately led to the hypothesis that newly identified U11 and U12 snRNAs serve in roles analogous to U1 and U2 during splicing (Montzka and Steitz, 1988). A role for U11 and U12 was confirmed *in vitro* (Tarn and Steitz, 1996a) and *in vivo* (Hall and Padgett, 1996; Kolossova and Padgett, 1997), and bolstered by the additional identification of snRNAs analogous to U4/U6, U4atac and U6atac (Tarn and Steitz, 1996b; Incorvaia and Padgett, 1998). Based on their relative abundance in analyzed genomes, the intron types and their respective spliceosomes were henceforward labeled major (U2-type) and minor (U12-type) in those eukaryotes that maintain them in parallel (Burge et al., 1998; Lynch and Richardson, 2002; Lin et al., 2010). Of note, major introns and the major spliceosome are ubiquitous in the eukaryotic lineage, while minor introns and the minor spliceosome are reportedly absent in some lineages, such as *Caenorhabditis elegans* (Burge et al., 1998).

Both the major and minor spliceosomes employ U5 snRNA, and each snRNA further associates with specific proteins in their splicing-competent forms (Tarn and Steitz, 1996a; Tarn and Steitz, 1997). Though the individual snRNAs have specific proteins associated with their regulation and maturation, many of the remaining proteins that comprise the spliceosome are shared between both the major and minor molecular machineries (Will et al., 1999); for a more comprehensive presentation of individual spliceosome components, see Olthof et al. (2022). Worth noting, the same protein can carry out different roles in each spliceosome, as is observed by URP (also called ZRSR2) (Tronchère et al., 1997; Shen et al., 2010). While the size and dynamic composition of the spliceosome can make it difficult to fully resolve, identifying the proteins involved in splicing regulation remains an area of active investigation. Recent biochemical and cryogenic electron microscopy investigations to this end have significantly enhanced our understanding of minor spliceosome-specific proteins. For example, the protein compositions of U4.U6/U5 and U4atac.U6atac/U5 tri-snRNP complexes were previously thought to be identical. However, co-immunoprecipitation and co-migration analyses have suggested that CENATAC may aid in 5′SS recognition for a subclass of minor introns characterized by AT-AN terminal di-nucleotides. Previously known as *CCDC84*, *CENATAC* was renamed following its mutagenic link to intron retention in human genes that contribute to chromosome stability and segregation (de Wolf et al., 2021). Interestingly, phylogenetic profiling of CENATAC across 90 eukaryotic species showed that it co-enriched with other components of the minor spliceosome, including the newly characterized SCNM1 protein (de Wolf et al., 2021). The U12 snRNA is flanked by the N-terminal C_2_H_2_ zinc fingers of SCNM1, which interacts with the U12/BPS duplex and the U12 Sm ring (Bai et al., 2021). The N-terminus of SCNM1 also functions to stabilize U6atac and RNF113A at the 5′SS, maintenance of which is required for spliceosome activation *in vivo* (Incorvaia and Padgett, 1998; Bai et al., 2021). Structural insights were also important in identifying the novel minor spliceosome protein, RBM48, which is now known to bind ARMC7 and interact with terminal ends of U6atac snRNA via conserved RNA binding residues (Bai et al., 2021; Siebert et al., 2022). Structural analyses of the minor spliceosome are a recent advancement and do not yet cover all phases of splicing, notably excluding the U11/U12 di-snRNP. As such, there remains the possibility for other unidentified components regulating the nuances of minor intron splicing.

**FIGURE 3:**
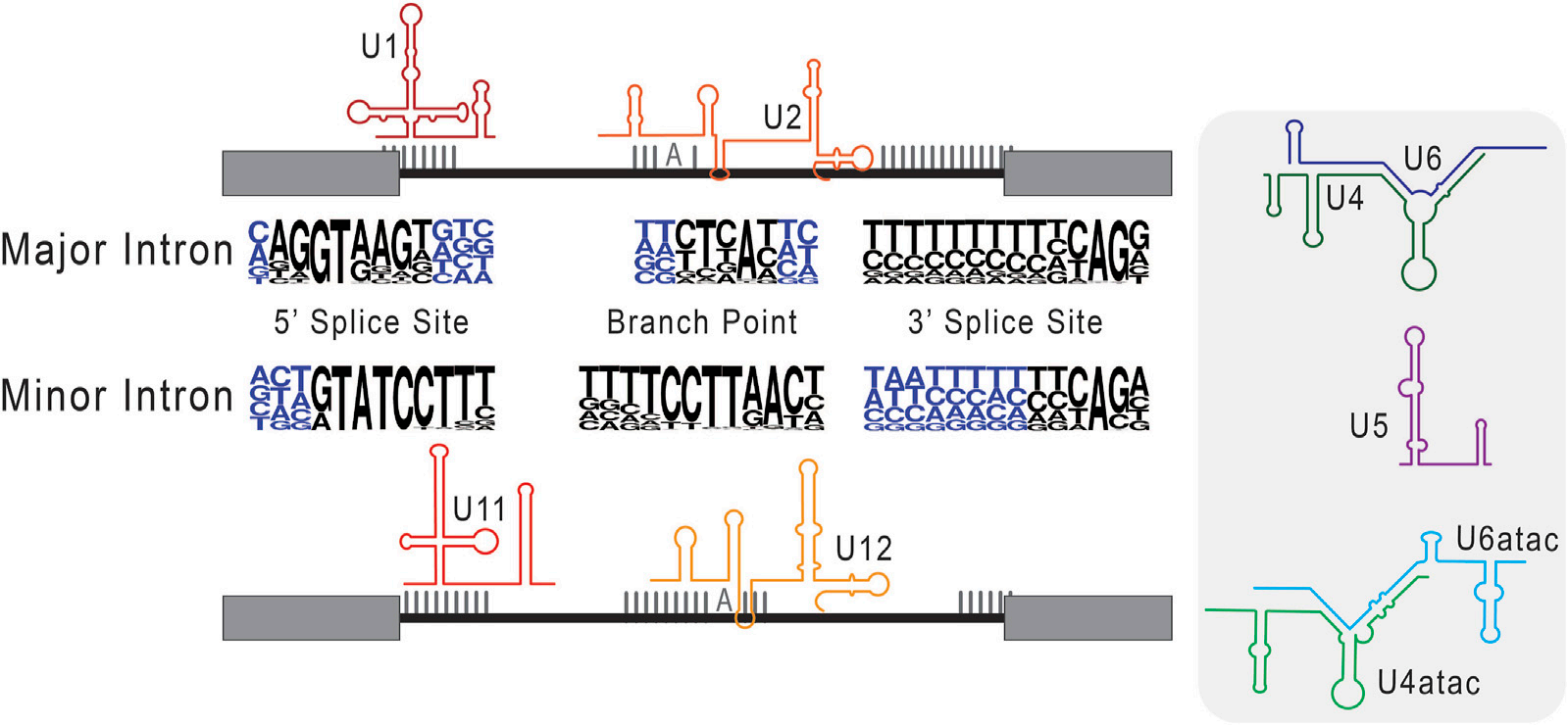
Consensus sequences used in the classification of major versus minor introns. Here, we schematize splice site selection by the respective components of the major and minor spliceosomes. The snRNAs of the major (U1 and U1) and minor (U11 and U12) spliceosome are shown base pairing to their cognate consensus sequences. In the center, next to the respective major intron and minor intron labels, we depict consensus sequences as nucleotide frequency plots. Here, the relative size of the nucleotide represents how frequently it is observed in that genomic position. Right of this schematic, we include the remaining core snRNAs that are unique to major (U4 and U6) and minor (U4atac and U6atac) intron splicing, as well as the shared U5 snRNA.

A delineation between major versus minor intron splicing is often based on the quantitative analysis of splice site conservation, and thus relative splice site strength. Intron splice sites are generally scored based on the degree of similarity to the major versus minor intron consensus sequences found in Figure 3, using position weight matrices (Sheth et al., 2006; Alioto, 2007; Olthof et al., 2019; Moyer et al., 2020). The resultant major or minor intron classification inherently dictates how we interpret its processing, such that bioinformatically classified minor introns are predicted to be spliced by the minor spliceosome, and *vice versa*. However, RNA sequencing data has shown that, upon inhibition of the minor spliceosome, not all bioinformatically classified minor introns show a splicing defect (Olthof et al., 2019). Thus, parallel existence of major and minor spliceosomes, combined with diverging intron consensus sequences, reveal an added complexity in the relationship between a given intron and its recruited spliceosome. Akin to how the concept of a single intron type was disrupted by the discovery of minor introns; it seems increasingly likely that the binary classification or major versus minor itself is insufficient to fully resolve all introns. Rather, evidence has begun to suggest that the stringency of the classification schema fails to consider the fluidity of exons and introns. For example, use of novel splice sites within exonic regions in the unicellular Paramecium is evidence of intronization activity in eukaryotes (Ryll et al., 2022). In essence, these findings increasingly suggest that the current approach to intron classification is too reductive to fully capture the complexities and dynamic regulation of eukaryotic introns. Towards this end, an examination of minor-type splice sites in Physarum polycephalum has suggested that minor introns may exist in divergent, if not degenerative, types (Larue et al., 2021) and this idea is currently being refined in other studies that combine principles of speciation and comparative genomics.

## How gene architecture informs splice site selection

Spliceosomal introns are known to range from tens of base pairs in length to hundreds of kilobases in length, with a mean length that is smaller in lower eukaryotes and larger in higher eukaryotes (Sakharkar et al., 2004; Piovesan et al., 2015; Abebrese et al., 2017; Li et al., 2017; Jakt et al., 2022). The size of an intron has an inherent impact on gene expression, as it will take longer for transcription machinery to create nascent transcripts. In turn, this will impact the kinetics of co-transcriptional intron splicing; these ideas have been explored in depth (Herzel et al., 2017; Wallace and Beggs, 2017; Neugebauer, 2019). It is long since established that relative intron and exon lengths can differentially affect splicing efficiency due to a presence or absence of regulatory elements and differing requirements for catalysis (Fox-Walsh et al., 2005; Kandul and Noor, 2009; Pai et al., 2017). Splicing efficiency refers to the proportion of spliced versus un-spliced transcripts relative to the number of total transcripts. This is commonly assessed using computational strategies that characterize splice events in the transcriptome (de Melo Costa et al., 2021; Jiang et al., 2023), followed by a validation of observed changes in expression using techniques such as RT-PCR. In one assessment of how splicing efficiency and gene expression patterns may be coupled, intron length was found to contribute to the temporal coordination that is required for co-expression of genes with interdependent biochemical functions (Keane and Seoighe, 2016). This idea is further reflected by distinct differences in splice site strength relative to intron length, and by differences in splicing efficiency and mRNA abundance relative to gene length (Gelfman et al., 2011; Sánchez-Escabias et al., 2022). Vertebrates are known to increase splicing efficiency around longer introns via cell-specific recursive splicing and transposable elements that form stems with intronic RNA loops to juxtapose splice sites (Shepard et al., 2009; Zhang et al., 2018). For details on recursive splicing, please see published reviews (Georgomanolis et al., 2016; Gehring and Roignant, 2021; Joseph et al., 2022; Pitolli et al., 2022).

Separate from this, longer introns may also have a propensity to contain multiple splice sites within one intronic feature, leading to alternative splicing from competing splice site use (Sun and Chasin, 2000; Roca et al., 2003; Kapustin et al., 2011). Meaning it becomes increasingly likely that multiple splice sites be present, in addition to exonic splicing enhancers and silencer elements, which themselves can act as determinants of splice site usage (Black, 2003; Wang et al., 2006). It thus follows that the sequence content of the intron to be excised can drive splicing progression. Splice site selection is thought to occur by competing intron- and exon-definition models, which describe how the spliceosome assembles either through cross-bridging interactions across the intron itself or across the flanking exon. Specifically, the intron-definition model refers to the mechanism whereby 3′ SS selection is informed by recognition of the upstream 5′SS, such that the spliceosome assembles across the intron. For exon-definition interactions, 3′ SS recognition depends instead on recognition of the downstream 5′SS (Robberson et al., 1990; Berget, 1995; Romfo et al., 2000; De Conti et al., 2013; Olthof et al., 2021). For example, most genes in *Saccharomyces cerevisiae,* contain only one, short intron. With this gene architecture, it is not surprising that intron-definition interactions predominate. Surprisingly, cryo-electron microscopy structures of the pre-catalytic spliceosome demonstrated that the same splicing machinery can perform exon-definition interactions in multi-intronic genes (Li et al., 2019). This finding brings to bear uncertainty as to how and when an intron- versus exon-centric model is utilized. This becomes especially important in higher vertebrates which have a larger intronic burden.

Reconciliation between the intron- and exon-definition models is coupled with new insight into how proximity rules inform splice site selection. Based on the length of an intron, the intron-centric proximity rule dictates a preference for the spliceosome to assemble over a splice site pair that minimizes the distance between 5′ and 3′ end selection (Reed and Maniatis, 1986). More recently, computational analyses by Carranza et al. (2022) refined the exon-centric proximity rule, by which splice sites are selected to minimize the exon-spanning distance. Meaning if one were to imagine an intron with two adjacent sets of 5′ and 3′ splice sites, the intron-centric proximity rule would employ the innermost splice site pair, maximizing the resultant exons. Meanwhile, the exon-centric proximity rule would, in contrast, use the exon-proximal splice sites to maximize the size of the intron being excised. In either case, commitment to the intron-centric or exon-centric proximity rule has commensurate intronization/exonization consequences as molecular machinery decides whether to select for the smaller or larger exonic sequences. In addition to intron size, studies suggest that GC content of the intron may also be a determinative factor in the mechanism employed for splice site selection. In one study, (Tammer et al. (2022), examined the nucleotide composition of exons versus introns and subsequently identified genes they refer to as “differential” or “leveled”. In “leveled” genes, GC content is found to be similarly high in exons and introns, while “differential” genes are ones wherein GC content is low in exons, and even lower in introns. Notably, Tammer et al. (2022), describe a partiality for intron-definition interactions across “leveled” genes, while exon-definition interactions predominate over “differential” genes. This finding is in line with previously reported links between differential GC content and splice site selection (Amit et al., 2012).

### Spliceosomal sRNAs

As described above, snRNAs confer the primary function of the spliceosome through formation of specific base pair interactions with consensus sequences in the intron. The presence and function of snRNAs is essential for recognition and restructuring of the nascent mRNA transcript in the sequential, exothermic transesterification reactions that constitute splicing.

In general, most snRNAs (U1, U2, U4, U5, U11, and U12) are transcribed by RNA polymerase II, while U6 and U6atac expression are largely dependent on RNA polymerase III (Reddy et al., 1987; Jawdekar and Henry, 2008; Younis et al., 2013). Initiation of transcription of these snRNAs is highly reliant on the proximal and distal sequence elements located upstream of the snRNA-encoding region. Specifically, because they serve as promoter and enhancer elements for recruitment of transcription machinery through interactions with the SNAPc transcription factor complex and stabilizing co-activators (Sadowski et al., 1993; Henry et al., 1998; Mittal et al., 1999; Dergai et al., 2018). Structural insights by cryogenic electron microscopy of SNAPc during the transcription of U6 has revealed the importance of conserved subunits which recognize and bind the proximal sequence element (Sun et al., 2022). One unique exception to this rule is for the expression of human U4atac snRNA, which is embedded into an intron of *CLASP1* (Edery et al., 2011). Therefore, U4atac expression relies on RNA polymerase II mediated transcription of this gene, as well as successful splicing of this intron.

Within the genome, spliceosomal snRNAs often exist both as gene copies and gene families, whereby divergent genes can encode for variant snRNAs with nucleotide polymorphisms (Denison et al., 1981; Abel et al., 1989). There are both productive and unproductive variants of the snRNAs annotated; productive snRNAs are capable of splicing, while those that are not are termed pseudogenic (Mabin et al., 2021). For example, the U6 snRNA has many pseudogenes and fewer productive copies that are dispersed throughout the genome, whereas U1 and U2 snRNAs are encoded by many functional copies that are organized in homogenous repeats (Van Arsdell and Weiner, 1984; Theissen et al., 1985; Tichelaar et al., 1998; Domitrovich and Kunkel, 2003; O’Reilly et al., 2013; Anjos et al., 2015). The presence of multiple gene copies may in part explain the splicing-independent roles of U1 and U2 in regulating transcription termination and 3′ end processing (Friend et al., 2007; Di et al., 2019; So et al., 2019). Moreover, the idea that multiple gene copies exist for minor spliceosomal snRNAs, including U4atac and U6atac, warrants further investigation. Even if multiple gene copies do exist, it must be noted that U6atac expression is maintained at a lower level through rapid post-transcriptional turnover (Younis et al., 2013).

Perhaps counterintuitively, U5 snRNA has the smallest gene family, yet it is the only shared snRNA between the major and minor spliceosomes. Investigations by Mabin et al. (2021) into the relevance of snRNA variants in splicing led to the discovery of high sequence identity between U5 variants. In fact, they report several U5 variants with a conserved stem consensus sequence (CUUUU) that can be incorporated into catalytic spliceosomes. Based on these observations, it has been suggested that U5 may not have a canonical snRNA; rather, specific variants may be optimal for use in one spliceosome type over the other (Mabin et al., 2021). While mechanistically unvalidated, this logic is consistent with the analogous nature of the other major versus minor snRNAs. Yet, it also remains possible that these U5 variants are regulated in a context-dependent way, as is observed for U1 snRNA variants during human stem cell programming (Vazquez-Arango et al., 2016). Additionally, U5 snRNA variants have been identified in regulating development in humans, *Drosophila,* and *Lytechinus variegatus* (Sontheimer and Steitz, 1992; Morales et al., 1997; Chen et al., 2005). The expression of snRNA variants to specify a differentiating transcriptome is not unique to U5 snRNA, but more broadly detected for other snRNAs and across species (Lo and Mount, 1990; Cáceres et al., 1992; Sierra-Montes et al., 2005; O’Reilly et al., 2013; Lu and Matera, 2014).

Functional sequence variants of the snRNAs have the potential to contact cryptic or degenerating splice sites, make novel protein interactions, and adopt secondary structures that alter spliceosome conformation. It is thus possible, given our evolving understanding of consensus sequences, that these variant snRNAs do confer complementarity to specific intron splice sites. Accordingly, from an intron-centric perspective, we must allow for the possibility that seemingly unproductive snRNAs are leveraged to splice a specific subset of introns. A role for non-consensus intron classes was voiced by Hudson et al. (2019), whose bioinformatics analyses of diplomonad and parabasalid lineage eukaryotes revealed splice site sequences that diverged from both the major and minor consensus sequences. They similarly identify divergent snRNAs, though they maintained key functional structures including stem loops and putative Sm binding sites. Perhaps more compelling, the discovered snRNAs showed aggregate features of both the major- and minor-type snRNAs, suggesting a propensity for the spliceosome to adopt complementarity to *trans-*spliced introns.

It remains to be established if variant snRNAs are evolutionarily selected for use in differential splicing or if they arise stochastically. Though, one could imagine that selective use of a variant splicing component would provide an opportunity to splice novel or divergent splice site sequences. It is known that mutations in the snRNAs can have pathogenic effects, as demonstrated by *RNU12* which is causal to early onset cerebellar ataxia (Elsaid et al., 2017). Additionally, snRNA secondary structure is important for splicing as it dictates the RNA-protein interactions necessary for spliceosome assembly. For example, U11/U12-65K binds the 3′ stem loop II (SLII) of U12 snRNA based on the integrity of this structure and its RNA binding motif. Further, 3’ truncation mutants that disrupt the U12 SLIII are targeted for degradation by the nuclear exosome targeting complex upon reimport to the nucleus (Norppa and Frilander, 2021). In another example, the U2/U6 and U12/U6atac complexes are remodeled and stabilized prior to the first catalytic step in splicing by intramolecular base pairing with RBM22 (Ciavarella et al., 2020). Regardless, developmentally regulated snRNA variants demonstrate that mutations outside of critical structures may maintain, albeit differential, functionality. Thus, it stands to reason that variant snRNAs without disease-causing consequences to splicing may have a context-dependent role in the regulation of introns with divergent consensus sequences.

### The evolutionary advantages of introns

Introns have served a valuable evolutionary role for eukaryotes in that they are more prone to genetic drift compared to exons. Introns appear to be under weaker selection than exons in somatic cells, which may be due to a mismatch repair system employed for exons that is notably lacking for introns (Hoffman and Birney, 2006; Resch et al., 2007; Frigola et al., 2017; Rodriguez-Galindo et al., 2020). Using a combinatorial multi-omics approach, Huang et al. (2018), has attributed the selective protection and mismatch repair of actively transcribed genes to an enrichment of H3K36me markers, which help regulate molecular responses to DNA damages induced by prolonged euchromatic conformation. More broad analyses of the differentiating human methylome reveal distinct differences in methylation pattern between genomics features, such that methylation is generally more common to exons than introns (Laurent et al., 2010). This unequal distribution may explain the higher frequency of mismatch repair observed for exons versus introns. In this capacity, introns can essentially act as a sponge to harbor mutations that would be otherwise detrimental in exonic sequences. Nevertheless, many mutations in intronic elements are linked to diseases, suggesting that there are limits to the number of mutations an intron can withstand. Mutations at splice sites and within introns are known to underscore an array of genetic and developmental disorders, including muscular dystrophy (Dominov et al., 2019) and inherited retinal diseases (Qian et al., 2021). Pathogenic disorders due to mutation of the spliceosome, i.e., spliceosomopathies, include but are not limited to craniofacial defects, myelodysplastic syndromes, and retinitis pigmentosa (Griffin and Saint-Jeannet, 2020). For review of major and minor splicing-associated diseases, see (Anna and Monika, 2018; Olthof et al., 2022).

**FIGURE 4:**
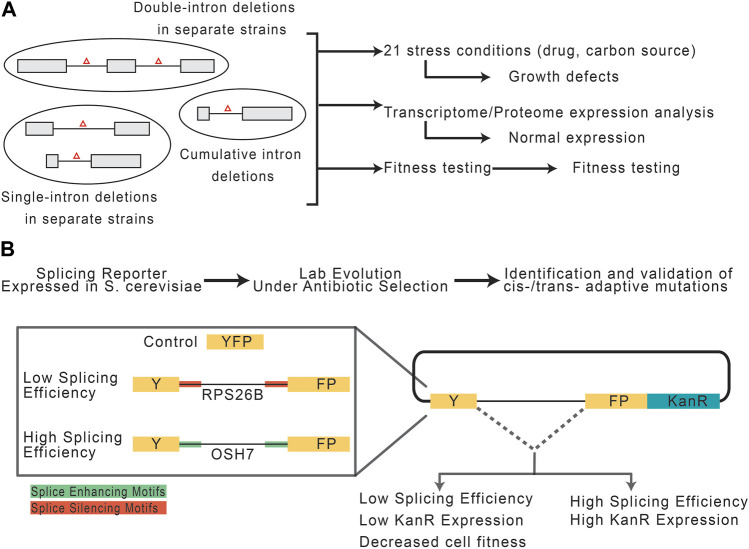
Potential role of introns and spliceosomal snRNAs in stress response. **(A)** Experimental paradigm, adapted from Parenteau et al. (2008), to assess the consequences of intron depletion in *S. cerevisiae.* Yeast with sets of removed intron(s) were grown under normal or stress conditions and assessed for fitness. **(B)** Experimental paradigm, adapted from Frumkin et al. (2019), to assess the capacity of introns and the splicing machinery to adapt to selective pressures.

While introns are seemingly advantageous, prokaryotes show that the absence of introns is not prohibitive to life. This begs the question, to what extent do eukaryotic cells really require introns? In one study, Parenteau et al. (2008), investigated the consequences of intron depletion in *Saccharomyces cerevisiae* (Figure 4A). Introns are far less abundant in *S. cerevisiae* compared to other species, such as vertebrates and land plants, making the yeast genome a strong model for intron depletion studies (Csuros et al., 2011)*.* Indeed, *S. cerevisiae* could survive without introns, however, intron-depleted strains fared variably when subjected to drug-induced and carbon source stresses. However, transcription machinery was found to be capable of responding to expression deficits following intron-depletion by using alternative promoter selections, highlighting the role introns play in expanding the eukaryotic transcriptome (Parenteau et al., 2008). Should one suppose that introns can be leveraged to induce stress-related patterns of gene expression, it then follows that the splicing efficiency of an intron is responsive to stress application. This idea was recently explored by Frumkin et al. (2019), who employed YFP reporter constructs containing known introns with high and low splicing efficiencies embedded and fused to a kanamycin resistance cassette (Figure 4B). To test the capacity of introns and the spliceosome to respond to metabolic pressure, the constructs were expressed in *S. cerevisiae* cells under antibiotic selection and subjected to a lab-evolution paradigm. Growth and transcriptomic analyses of derived cell generations revealed independent, adaptive mutations occurring both cis- and trans-to improve splicing efficiency and thus antibiotic resistance and cell survivability. The cis-mutations were proposed to increase accessibility of splice site sequences, while trans-mutations might increase the cellular abundance of splicing machinery. Importantly, cis-fitness-inducing mutations could alleviate selection-independent splicing inefficiencies, however, mutations in trans-were particularly advantageous during periods of active selection (Frumkin et al., 2019). Though these experiments were performed in *S. cerevisiae*, one can imagine that similar mechanisms may be employed for evolutionary adaptation. For example, in ecotypic Cichlid fish, alternative splicing is a dominant mechanism for rapid changes in gene expression. Specifically, alternative splicing underpins the diversification of jaw morphology as it relates to the food they have evolved to consume in different ecological niches (Singh et al., 2017).

### The influence of introns on gene expression

In both mammals and plants, the presence of introns is known to enhance gene expression in a phenomenon sometimes referred to as intron-mediated enhancement (Brinster et al., 1988; Furger et al., 2002; Samadder et al., 2008). The recent development of sequencing techniques such as GRO-seq, mNET-seq and long read sequencing have revealed that splicing of neither major nor minor introns occurs in isolation, but rather in a highly active genomic context where splicing and transcription are coupled both kinetically and physically (Nojima et al., 2015; Herzel et al., 2017; Sheridan et al., 2019; Drexler et al., 2020; Reimer et al., 2021; Zhang et al., 2021). In the context of splicing informing transcription, the position of the intron matters, as promoter-proximal introns are especially known to enhance transcription (Furger et al., 2002; Rose et al., 2008). The knowledge that introns may enhance transcriptional output was leveraged to modify the generally used CMV promoter for expression plasmids, whereby introduction of an intron significantly upregulated transcription of downstream coding sequences (Simari et al., 1998).

The mechanism by which 5’ introns regulate transcription involves, at least in part, control of the open chromatin signatures H3K4me3 and H3K9ac, which facilitate recruitment of RNA polymerase II and general transcription factors to promoters. These marks are deposited at the first exon-intron boundary of genes, explaining how the distance between transcription start site and the first intron can influence the expression level of a gene (Bieberstein et al., 2012, Lister, 2009). Interestingly, differential methylation patterns are not unique to protein-coding genes, as revealed through a bioinformatics model which considered the modified human nucleosome library and analysis of splicing efficiency. For example, high nucleosome density was observed in the internal exons of long non-coding RNAs, while high H3K4me3 signals were observed in upstream introns. Importantly, these signatures were often associated with exon skipping and intron retention, particularly around the first intron (Dey and Mattick, 2021). While a tissue-independent model likely obscures some of the nuanced features regulating splicing-dependent gene expression, a genome-wide comparative analysis by Anastasiadi et al. (2018) revealed that correlation between CpG methylation and gene expression is unique to the first exon and intron. As CpG markers of DNA methylation tend to decrease across exons and increase across introns, it is possible that methylation may inform gene expression by mediating intron splice site recognition (Laurent et al., 2010). In fact, removal of promoter-proximal introns altogether reduces levels of H3K4me3 and chromatin-bound RNA polymerase II, reducing transcriptional output (Bieberstein et al., 2012; Laxa et al., 2016). Similarly, reduction in chromatin accessibility was observed when formation of the active spliceosome was inhibited by spliceostatin A. This finding highlights an important role for the spliceosome in regulating transcriptional output. Notably, this effect was not intrinsic to the presence of introns, but dependent on their splicing (Bieberstein et al., 2012).

One caveat to the spliceostatin A experiment is that it inhibits the entire splicing machinery, without revealing the specific interactions between the spliceosome and intron consensus sequences that enhance transcription. In fact, it is not the entire spliceosome that needs to be activated for transcription enhancement, as the formation of stable interactions between U1 snRNA and the promoter-proximal 5′SS can enhance transcription (Engreitz et al., 2014). Recruitment of the U1 snRNP to the first intron enhances transcription initiation through recruitment of general transcription factors, such as TFIIH, and stabilization the first formed phosphodiester bond by RNA polymerase II (Kwek et al., 2002; Damgaard et al., 2008). Notably, this effect is independent of its role in major intron splicing, as mere introduction of a 5′SS sequence is sufficient to enhance transcription (Damgaard et al., 2008). This splicing-independent function of U1 might help explain its constitutive association with the elongating RNA polymerase II and why it is likewise recruited to intronless genes (Spiluttini et al., 2010; Leader et al., 2021).

Beside the role of U1 in transcription initiation, U1 snRNA is also independently involved in preventing pre-mature transcription termination, which can occur if RNA polymerase II encounters a polyadenylation site within an intron. Surmounting 3′ end sequencing data has revealed that introns often contain cryptic or pre-mature polyadenylation sites that result in the destabilization of RNA polymerase II, thereby producing truncated transcripts incapable of encoding a protein (Di Giammartino et al., 2011). Remarkably, the production of these truncated transcripts can be blocked by the U1 snRNA in a process called telescripting. In this capacity, U1 is capable of complexing with 3′ processing factors to protect the mRNA from premature cleavage and termination (Kaida et al., 2010; Berg et al., 2012). This mechanism occurs alongside the elongating polymerase to allow for U1-mediated suppression of cryptic polyadenylation sites in the intron or 3′ UTR (Di et al., 2019). Proper transcription termination is important in regulating the length and structure of the 3’ UTR, which in turn promotes formation of the export-competent messenger ribonucleoprotein. Similar to U1, U11 is expressed more highly than is necessary for its function in splicing (Baumgartner et al., 2015). Given that U11 is more abundant than U12 though they present at the same stoichiometric ratio within the minor spliceosome, U11 may similarly have splicing-independent functions. We speculate that U11 may either function in a mechanism like telescripting or participate in an alternative function, such as the subnuclear clustering of expressed minor intron-containing genes.

### Localization of spliceosome components

Genes, chromatin, and RNA polymerase II have a subnuclear organization around topologically-associated domains to phase-separate euchromatic regions of active transcription (Szentirmay and Sawadogo, 2000; Ulianov et al., 2016; Szabo et al., 2020). Alongside this, it would be reasonable to hypothesize that splicing machinery is also organized to support efficient gene expression. In fact, major and minor spliceosome snRNPs display similar partiality for nuclear localization, except for U6 and U6atac snRNPs (Spiller et al., 2007; Pessa et al., 2008; Steitz et al., 2008). In the nucleus, matured snRNPs of the major spliceosome accumulate in phase-separated speckles that serve to organize spliceosome components adjacent to perichromatin regions of active transcription. This was concluded following nonradioactive and fluorescence *in situ* hybridization analyses, as well as RNA and protein blotting of subcellular compartment extracts (Pessa et al., 2008). While this model is an enticing way to interpret speckles as a regulatory mechanism over major intron splicing, it does not necessarily extend to that of minor introns. Given that the major and minor spliceosomes are known to interact with each other in the splicing of minor intron-containing genes, the model does not encompass all mechanisms of splicing (Akinyi and Frilander, 2021; Olthof et al., 2021). Punctate subcellular localization of spliceosome machinery is not specific to core snRNP components, but also includes some of the auxiliary splicing factors that contribute to spliceosome stability, conformational changes, and catalytic activity during splicing. These non-snRNP factors are integral to spliceosome assembly and the coordinated action of snRNPs during splicing (Bindereif and Green, 1990). For example, a new model supposes that the unequal phasic separation of SR proteins and heterogenous nuclear ribonucleoproteins proteins (hnRNP) at nuclear speckles can contribute to splice site selection. Specifically, the positional distribution of SR proteins and hnRNPs around a splice site generally determines the positive or negative regulatory effect of their binding, and taken with their distinct subnuclear distributions, can dictate the use of splice sites (Liao and Regev, 2021).

In all, here through an intron-centric lens, we focus our attention on the myriad of regulatory and functional consequences that have emerged by the presence of introns in the genome. Thus, we hope that future studies will begin to shed light on this “dark matter” of the eukaryotic genome to uncover the secrets buried within. Importantly, the advent of next-generation sequencing and computational analysis will invariably play a critical role in uncovering some of these mysteries. Throughout this article, we have described several of these methods, and here we point readers to other reviews (Halperin et al., 2021; Lorenzi et al., 2021; Gondane and Itkonen, 2023).
